# Prostaglandin profiling reveals a role for haematopoietic prostaglandin D synthase in adipose tissue macrophage polarisation in mice and humans

**DOI:** 10.1038/ijo.2015.34

**Published:** 2015-04-21

**Authors:** S Virtue, M Masoodi, B A M de Weijer, M van Eijk, C Y L Mok, M Eiden, M Dale, A Pirraco, M J Serlie, J L Griffin, A Vidal-Puig

**Affiliations:** 1 University of Cambridge Metabolic Research Laboratories, Wellcome Trust-MRC Institute of Metabolic Science, Cambridge, UK; 2 Medical Research Council Human Nutrition Research, Cambridge, UK; 3 Nestlé Institute of Health Sciences, Lausanne, Switzerland; 4 Department of Endocrinology and Metabolism, Academic Medical Centre, University of Amsterdam, Amsterdam, The Netherlands; 5 Department of Medical Biochemistry, Academic Medical Centre, University of Amsterdam, Amsterdam, The Netherlands; 6 Metabolomx, Mountain View, CA, USA; 7 The Department of Biochemistry, Cambridge, UK

## Abstract

**Background/Objectives:**

Obesity has been associated with both changes in adipose tissue lipid metabolism and inflammation. A key class of lipid-derived signalling molecules involved in inflammation are the prostaglandins. In this study, we aimed to determine how obesity affects the levels of prostaglandins within white adipose tissue (WAT) and determine which cells within adipose tissue produce them. To avoid the effects of cellular stress on prostaglandin levels, we developed a multivariate statistical approach in which metabolite concentrations and transcriptomic data were integrated, allowing the assignment of metabolites to cell types.

**Subjects/Methods:**

Eicosanoids were measured by liquid chromatography-tandem mass spectrometry and mRNA levels using real-time PCR. Eicosanoid levels and transcriptomic data were combined using principal component analysis and hierarchical clustering in order to associate metabolites with cell types. Samples were obtained from C57Bl/6 mice aged 16 weeks. We studied the ob/ob genetically obese mouse model and diet-induced obesity model. We extended our results in mice to a cohort of morbidly obese humans undergoing bariatric surgery.

**Results:**

Using our modelling approach, we determined that prostglandin D_2_ (PGD_2_) in adipose tissue was predominantly produced in macrophages by the haematopoietic isoform of prostaglandin D synthase (*H-Pgds*). Analysis of sub-fractionated WAT confirmed that *H-Pgds* was expressed in adipose tissue macrophages (ATMs). Furthermore, *H-Pgds* expression in ATMs isolated from lean and obese mice was consistent with it affecting macrophage polarisation. Functionally, we demonstrated that H-PGDS-produced PGD_2_ polarised macrophages toward an M2, anti-inflammatory state. In line with a potential anti-inflammatory role, we found that *H-PGDS* expression in ATMs was positively correlated with both peripheral insulin and adipose tissue insulin sensitivity in humans.

**Conclusions:**

In this study, we have developed a method to determine the cellular source of metabolites within an organ and used it to identify a new role for PGD_2_ in the control of ATM polarisation.

## Introduction

As the world wide epidemic of obesity continues, a key question is how obesity leads to metabolic complications, including diabetes and cardiovascular disease. One proposed link between obesity, insulin resistance, diabetes and cardiometabolic complications is the concept that a loss of adipose tissue function leads to ectopic deposition of lipids in other organs, which in turn causes insulin resistance via the process of lipotoxicity.^1^ Understanding how obesity leads to adipose tissue dysfunction is therefore essential in order to design rational strategies for alleviating obesity-related metabolic complication. One class of molecules that has attracted considerable interest in the control of adipose tissue function are prostaglandins. Prostaglandins, which are derived from 20-carbon length polyunsaturated fatty acids, have been suggested to link the consumption of lipid-rich diets with adipose tissue dysfunction.

Although considerable research has focussed on the role of prostaglandins within adipose tissue, the principal focus of these studies has been on adipogenesis and adipocyte function,^2, 3, 4, 5, 6, 7, 8, 9^ rather than the immune compartment. In terms of adipocyte function, it is notable that many of the results observed for prostaglandins *in vitro* have been poorly recapitulated *in vivo*. For example, prostaglandin E_2_ (PGE_2_) has been demonstrated to inhibit lipolysis *in vitro* via the PTGER3 receptor.^4, 6^ *In vivo*, loss of PGE_2_ signalling via the PTGER3 receptor has been reported to cause lipodystrophy due to uninhibited lipolysis;^10^ however, another report demonstrated that *Ptger3* receptor KO mouse is actually obese,^11^ casting doubt on the importance of lipolytic regulation by this pathway *in vivo*.

In terms of eicosanoid levels in adipose tissue, several studies have been published investigating the effect of ω-3 fatty acid supplementation on lipid mediator levels in white adipose tissue (WAT)^12, 13, 14^ as well as traditional dietary models of diet-induced obesity using lard-rich diets^14, 15^ and the genetically obese db/db model.^14^ Intriguingly, in models of high-fat diet (HFD)-induced obesity, two-series prostaglandins were also reported to be downregulated, despite the presence of large quantities of linoleic acid (the precursor of arachidonic acid) being loaded into the system, at least in the case of prolonged dietary treatments.^14, 15^

The discrepancies between *in vitro* and *in vivo* effects of prostaglandins on adipose tissue function can be in part explained by the fact that many studies *in vitro* focus on adipocytes and preadipocytes, while *in vivo* adipose tissue is made up of many different cell types, which can both produce prostaglandins and respond to these signalling molecules. One of the most important cell types for mediating the metabolic phenotype of adipose tissue *in vivo* are macrophages.

Obesity in both mice and humans is characterised by an increase in both the number of macrophages and by a switch in macrophage polarity from an anti-inflammatory (M2) to an inflammatory (M1) state. Both infiltration of adipose tissue by macrophages^16^ and their polarisation to an inflammatory state have been shown to impact on whole organism insulin sensitivity.^17, 18, 19, 20^ In the past few years, multiple additional pathways regulating adipose tissue macrophage (ATM) polarisation have been investigated, implicating a diverse range of biological processes, including traditional inflammatory signalling pathways involving cytokines,^20, 21, 22^ cytokine signalling pathways,^21, 22, 23,24^ transcription factors involved in inflammatory signalling,^25, 26^ fatty acids^27, 28, 29, 30^ as well as physiological interventions such as exercise.^31^ However, despite this research the signal that initiates macrophage polarisation in genetic and diet-induced obesity remains to be fully elucidated.

Despite the growing body of evidence for the importance of macrophages to the control of adipose tissue function, the role of macrophages as a source of lipid mediators within adipose tissue remains relatively unexplored. One of the major issues for assessing the role of eicosanoids in specific cell types within any tissue is that mechanical isolation of cells induces cellular stress, almost certainly changing the endogenous levels of these molecules. To avoid the problem of cellular isolation stress, in this study we integrated prostaglandin levels from whole WAT with mRNA markers of different cell types and biological processes in a multivariate statistical model. Our model allowed the assignment of prostaglandins to specific cellular compartments within adipose tissue. Using this method, we investigated different genetic and dietary states of obesity and demonstrate that prostglandin D_2_ (PGD_2_) is predominantly produced by ATMs and upregulated in obesity. Using bone marrow-derived macrophages (BMDMs), we showed that haematopoietic isoform of prostaglandin D synthase (H-PGDS)-derived PGD_2_ acts during differentiation to generate macrophages that exhibit an anti-inflammatory M2 phenotype. Of relevance, *H-PGDS* expression in human subcutaneous WAT (scWAT) macrophages was positively correlated with both adipose tissue and peripheral insulin sensitivity.

## Materials and Methods

### Animal care and diets

Mice were housed at a density of four animals per cage in a temperature-controlled room (20–22 °C) with 12-h light/dark cycles. Wild-type (WT) C57Bl/6 and ob/ob mice were purchased from Charles River (Harlow, UK). Food and water were available *ad libitum* unless noted. All animal protocols used in this study were approved by the UK Home Office and the University of Cambridge. Animals were fed on a normal chow diet (SDS RM3 11.5% calories-from-fat) unless otherwise stated. HFD studies used 60% calories-from-fat diets (D12451 Research Diets, New Brunswick, NJ, USA). Genders of mice are stated in the figure legends.

### Eicosanoid extraction and quantification

Extraction of eicosanoids was carried out as described previously^20, 32^ with the following modifications. Prior to the acidification step and solid phase extraction, WAT was ground on liquid nitrogen and then homogenised in 15% (v/v) methanol in water using a FastPrep-24 tissue homogeniser (MP Biomedicals, Cambrigde, UK), followed by the addition of internal standard PGB_2_-*d*4 (40 ng) (Cayman Chemicals, Ann Arbor, MI, USA). Homogenates (1 ml) where transferred to a clean 2-ml Eppendorf tube (Starlab, Milton Keynes, UK), and a further 1 ml of 15% (v/v) methanol in water was added to the homogeniser tubes and the process repeated to extract any remaining eicosanoids. This second 1 ml was added to the Eppendorf tubes, and the total homogenate was centrifuged at 16 000 *g* for 5 min at 4 °C. The 'infranatant' between the lipid layer and the cellular debris was removed and put in a 15-ml pyrex tube. A further 1 ml of 15% (v/v) methanol in water was added to the remaining cellular debris in the Eppendorf, vortexed and centrifuged at 16 000 r.p.m. for 5 min. For details of the chromatography and mass spectrometry analyses, see Supplementary Information.

### Quantitative, real-time PCR and western blotting

See Supplementary Information for details.

### Subcellular fractionation of adipose tissue

Adipocytes, macrophages and stromal vascular fraction were isolated by collagenase digest followed by CD11b magnetism-activated cell sorter (Miltenyi Biotech, Bisley, UK) separation (see Supplementary Information for details).

### BMDM culture

BMDMs were generated from murine hind-limb bone marrow and differentiated in the presence of macrophage colony-stimulating factor from L929 cells. BMDMs were treated during differentiation with 10 μM HQL-79, 1 μM Ramatroban, a combination of 10 μM HQL-79 and 1 μM PGD_2_ or vehicle during differentiation. Macrophages were polarised using either lipopolysaccharide (100 ng/ml) or interleukin-4 (10 ng/ml).

### Human studies

Subcutaneous adipose tissue biopsies were collected from 17 individuals undergoing bariatric bypass surgery. Macrophages were extracted as described for mice (see Supplementary Methods). Prior to surgery, subjects underwent a two-step hyper-insulinaemic euglycaemic clamp. For details of the clamp subjects see de Weijer *et al.*,^33^ and for details of the clamp conditions see Supplementary Information.

### Statistics

Comparisons between mouse groups were carried out by one-way analysis of variance followed by Tukey's *post-hoc* test. For analysis comparing expression levels in adipose tissue fractions, pairwise comparisons were carried out within individual fractions. Pearson correlations were used for regression data. All statistics were carried out using SPSS 21 (IBM, Armonk, NY, USA). Principal component analysis (PCA) plots were generated using SIMCA-P+ 12.0.1 (Umetrics, Umeå, Sweden). For details of NEATmap generation, see Supplementary Information.

## Results

### Prostaglandin levels in WAT

**Figure 1:**
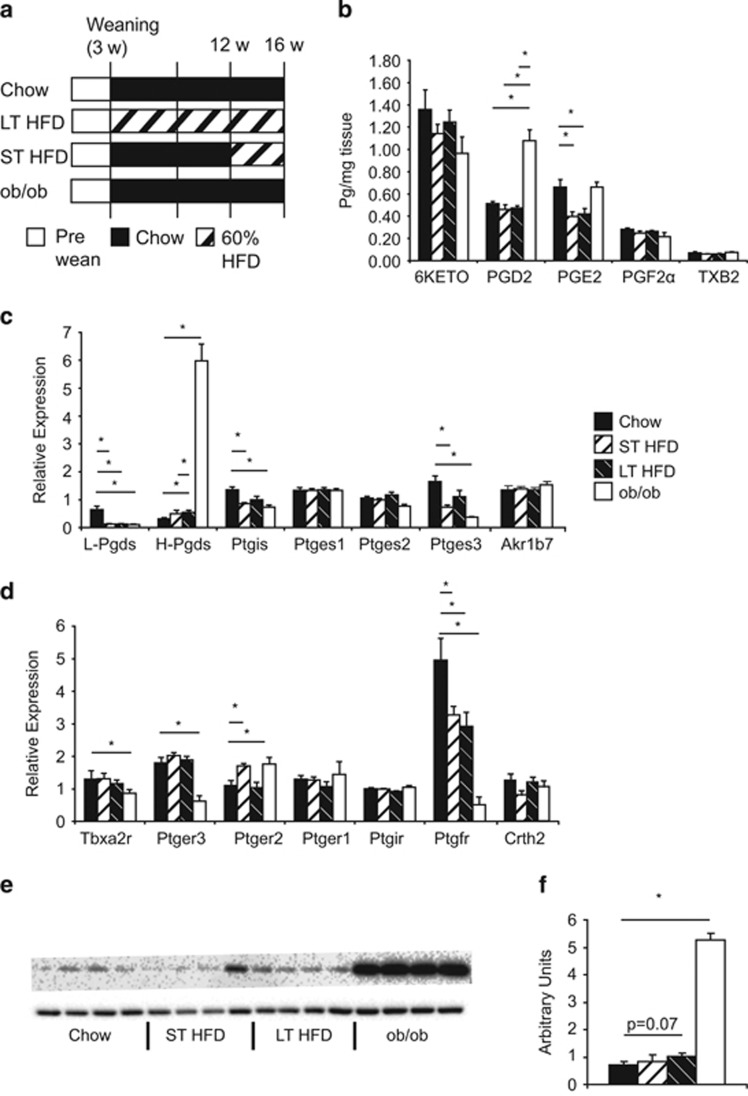
Levels of eicosanoids in WAT. (**a**) Diagram representing the different dietary and genetic models of obesity used. (**b**) Levels of cyclooxygenase products. (**c**) Expression levels of prostaglandin synthases. (**d**) Expression of prostaglandin receptors. (**e**) Representative western blotting of H-PGDS. (**f**) Quantification of H-PGDS protein in all samples normalised to β-actin. *N*=8 mice per group, C57Bl/6 females, 16 weeks of age. Chow, 16-week-old mice fed a chow diet from weaning; ST HFD, mice fed a HFD from 12 to 16 weeks of age; LT HFD, mice fed a HFD from weaning until 16 weeks of age; ob/ob, leptin-deficient mice fed a chow diet from weaning until 16 weeks of age. *P<0.05.

We used four separate mouse models to investigate the effects of obesity on adipose tissue prostaglandin profiles, with all mice culled at 16 weeks of age. Firstly, we used chow fed mice as a control. Second, we used ob/ob mice fed a chow diet as a model of extreme obesity without the effects of a change in diet. Finally, we used two mouse models of HFD feeding, short-term HFD mice were fed HFD from 12 to 16 weeks of age, whereas long-term HFD mice were fed HFD from weaning (3 weeks) until 16 weeks of age. The dietary conditions are shown in Figure 1a. As expected, the ob/ob mice were much heavier than chow or high-fat-fed WT mice and exhibited elevated blood glucose. Both the short-term ang long-term high-fat fed mice showed modestly increased body weight and increased blood-glucose levels compared with chow-fed controls (Supplementary Figure S1).

Analysis of prostaglandin concentrations from WAT revealed group-specific changes in PGE_2_ and PGD_2_. For abbreviations for prostaglandins and their receptors see Supplementary Table S1. PGE_2_ concentrations were reduced by high fat feeding, but not changed in the ob/ob model relative to WT controls, whereas PGD_2_ concentrations were increased in the ob/ob model compared with the other groups (Figure 1b).

### Expression of prostaglandin synthases and receptors

The mRNA expression levels of *H-Pgds* and *Ptgis* were regulated in a manner that resembled their cognate metabolites. Conversely, PGE_2_ metabolite concentrations were not similar to the expression profiles of any PGE_2_ synthases (Figure 1c). We measured the prostaglandin synthase *Akr1b7*,^34, 35, 36^ which has been shown to regulate adiposity,^37^ but detected no significant changes on a whole tissue level. Equally, thromboxane synthase was undetectable in adipose tissue and is likely to be expressed in platelets and red blood cells, which lack mRNA.

The receptors for most of the prostaglandins showed only limited regulation; however, the PGE_2_ receptor *Ptger3* was found to be downregulated in ob/ob mice on both an mRNA and protein level (Figure 1d and Supplementary Figures S6A and B), whereas the PGF_2_α receptor *Ptgfr* was found to be downregulated by both dietary conditions and genetic obesity on an mRNA level (Figure 1d) and by short-term HFD and in the ob/ob model on a protein level (Supplementary Figures S6A and B).

### Determining the cellular origin of cyclooxygenase metabolites

Adipose tissue is composed of multiple cell types. By using multivariate statistical analysis, coupled with mRNA markers of different cell types and biological processes, we attempted to assign specific metabolites to cell types and functions. We selected genes that are involved in several of the major biological processes that occur in adipose tissue in which prostaglandins have been implicated, including lipolysis^4, 6^ (PGE_2_) and adipogenesis (PGI_2_ and PGF_2_α).^2, 3, 5, 7, 8^ For a list of genes selected and their roles in specific processes in adipose tissue see Supplementary Table S2. Furthermore, we chose markers of preadipocytes, adipocytes, T-cells and macrophages, representing many of the major cell types located within adipose tissue that have clearly defined, discrete mRNA markers.

**Figure 2:**
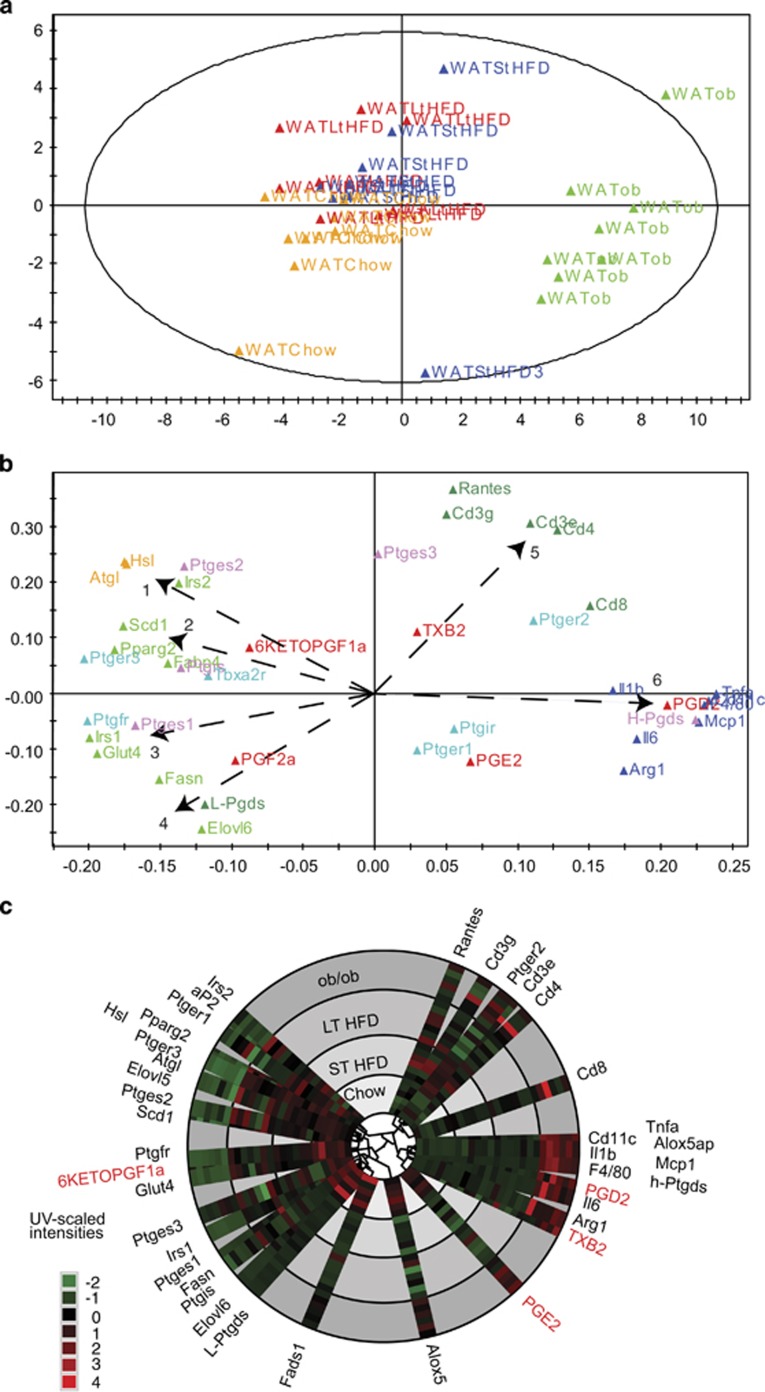
Clustering of prostaglandins with markers of biological processes. (**a**) PCA plot showing separation of WT, HFD fed and ob/ob mice based on prostaglandins and gene expression markers. (**b**) Loadings plot for PCA analysis shown above. Arrows show directions of contributions by: (1) Lipolytic markers, (2) adipogenic markers, (3) Insulin-sensitivity markers, (4) *De novo* lipogenic markers, (5) T-cell markers, and (6) Macrophage markers. (**c**) NEATmaps of transcriptomic and prostaglandin data. *N*=8 mice per group, for PCA analyses. *N*=6 mice per group for clustering analyses. C57Bl/6 females, 16 weeks of age.

We constructed a PCA model containing data from 32 mice and including expression from 32 genes (Figure 1 and Supplementary Figure S1). The model had a cumulative *R*^2^ score of 0.738 (Figure 2a). Although PCA is unsupervised, visual inspection of the PCA plots suggested that the first component (*x* axis) was driven by insulin sensitivity and the second component (*y* axis) appeared to be driven mainly by diet (Figure 2a).

The loading coefficients for the PCA plot (Figure 2b) revealed that markers of the same biological processes were closely related. Macrophage markers were the strongest driver of the separation of the ob/ob group (arrow 6). T-cell markers separated both the diet and ob/ob groups (arrow 5). The strongest drivers toward the insulin-sensitive chow group were *Glut4* and *Irs1* (arrow 3). The *de novo* lipogenic programme was suppressed in the ob/ob group. However, *Scd1* was associated with mice fed a HFD, whereas *Elovl6* and *Fas* were suppressed by high-fat feeding (arrow 4). Overall, the data demonstrated that the PCA model separated the animals based on gene expression profiles that correspond to known phenotypic changes associated with both the ob/ob model and high-fat feeding.

### Prostaglandin synthases and metabolites associate with specific biological processes

PGD_2_ was found to be closely associated with macrophage markers, as was *H-Pgds* (Figure 2b). Conversely, Lipocalin prostaglandin D synthase (*L-Pgds)*, the other PGD_2_ synthase, was not associated with PGD_2_ and is known to be a weak PGD synthase,^38^ suggesting that it was not the source of PGD_2_ in adipose tissue. 6-Keto-PGF_1α_ (a surrogate for PGI_2_) and its synthase *Ptgis* drove separation of the groups in the same direction as *Fabp4* and *Pparγ2*, in agreement with its known pro-adipogenic role. PGF_2_ was found to be driving separation in the same direction as *Fas* and *Elovl6*, two members of the lipogenic programme. We also, in a separate analysis, measured endothelial markers to determine whether any prostaglandins were associated with the endothelium (Supplementary Figures S5B and C), which suggested that the endothelium was not a major source of prostaglandins within adipose tissue.

Although PGE_2_ levels were not found to have any positive associations with biological processes studied, negative associations in PCA loading plots are found opposite to a given marker. *Hsl* and *Atgl* were located nearly diametrically opposite PGE_2_ on the loading plot, showing that PGE_2_ levels were negatively associated with lipolytic markers (Supplementary Figure S2A). Finally, PGE_2_ concentrations were negatively correlated pairwise with the expression of *Atgl* and *Hsl* (Figure 2b).

### Cluster analysis and circular heat map representation

To elucidate associations between metabolite concentrations and gene expression data in more detail, we used a heat map approach that combines hierarchical cluster analysis with a circular representation of the profiles.^39^ Using this combined visualisation approach, we detected that some metabolites and gene expression profiles showed clear overlap with the phenotypic groups. On the right hand side of the circular plot, we found profiles that showed a distinct upregulation in the ob/ob group while being downregulated in all the other phenotypic groups. This second approach again demonstrated the strong association between PGD_2_, *H-Pgds* expression and the macrophage compartment (Figure 2c).

### Expression of prostaglandin synthases in adipose tissue cellular compartments

**Figure 3:**
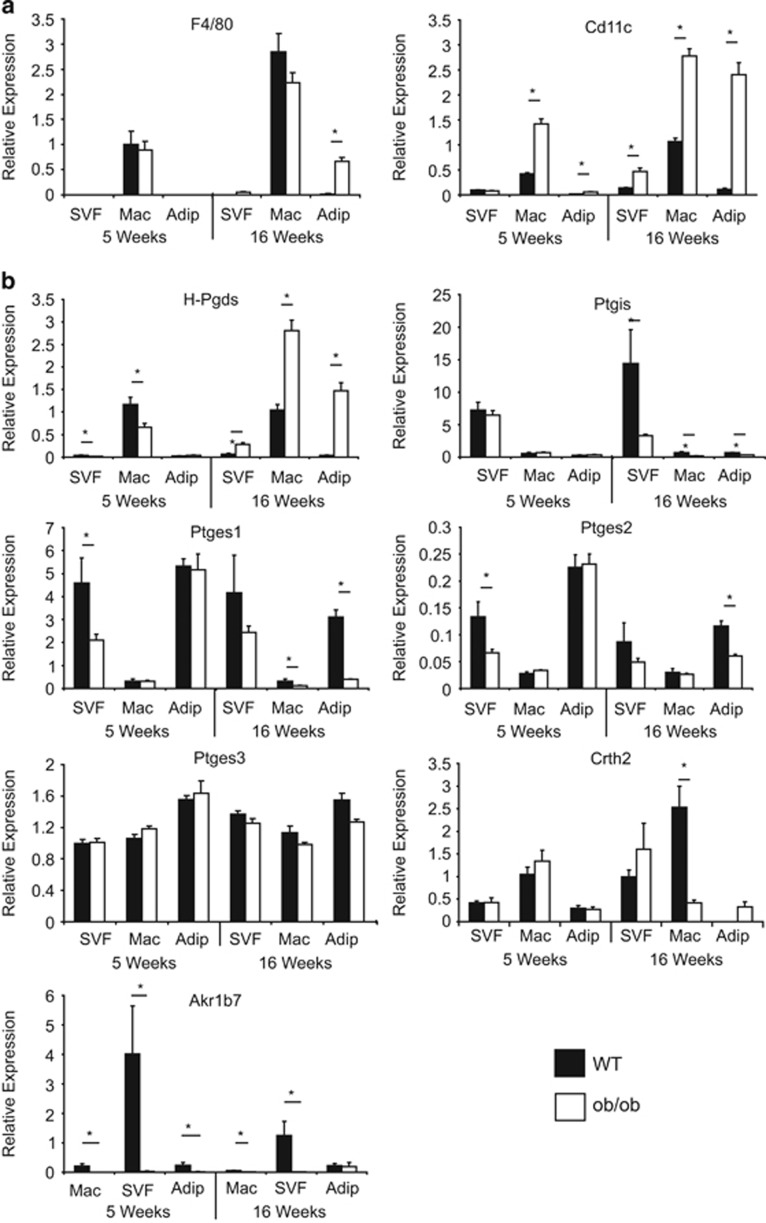
Expression profiles from sub-fractionated WAT. (**a**) Markers of macrophage abundance and polarisation. (**b**) Prostaglandin synthases detectable in WAT and the *Crth2* PGD_2_ receptor. *N*=>5 groups for each age and genotype, C57Bl/6 male mice. *P<0.05.

In order to substantiate the cell-specific origin of, and the processes that metabolites were assigned to, we next investigated the expression of prostaglandin synthases in macrophages isolated from the adipose tissue of 5- and 16-week-old WT and ob/ob mice. *Ptgis* was found to be predominantly expressed in the stromal vascular fraction and significantly downregulated in obese mice at 16 weeks (Figure 3b), in line with the reduced rates of adipogenesis in 16-week-old ob/ob mice. PGE synthases showed a varied pattern of expression. At 5 weeks, *Ptges1* and *Ptges2* were downregulated in the stromal vascular fraction in ob/ob mice compared with WT. At 16 weeks, *Ptges1* and *2* were downregulated in adipocytes and *Ptges1* was downregulated in the macrophages of ob/ob mice compared with controls.

### Validation of PGD_2_ as a macrophage-derived eicosanoid

In line with its association with the macrophage compartment in our multivariate statistical models, *H-Pgds* was found to be expressed predominantly in the macrophage fraction (Figure 3b). A second more comprehensive cellular isolation revealed that *H-Pgds* was expressed in macrophages at a higher level than other immune cells and was not expressed in the endothelial cells (Supplementary Figure S5A). Although *H-Pgds* appeared to be expressed in adipocytes isolated from 16-week-old ob/ob mice, this apparent expression probably represented contamination from macrophages, as *Cd11c* and *F4/80* (Figure 3a) as well as many other macrophage markers have been reported to be ‘expressed' in adipocytes.^40^ Further, on a protein level, H-PGDS was only detectable in ATMs in 16-week-old WT mice (Supplementary Figure S3C). It was notable that *H-Pgds* was significantly downregulated in ob/ob ATMs at 5 weeks and upregulated more than twofold in ob/ob ATMs at 16 weeks. We have previously reported a biphasic pattern of expression for multiple genes in macrophages from ob/ob mice, consistent with a switch from M2 to M1 polarisation.^40^ To further substantiate a role for *H-Pgds* in adipose tissue function, *H-Pgds* expression was profiled in multiple metabolic tissues. *H-Pgds* was expressed at higher levels in WAT than in the brown adipose tissue, skeletal muscle, heart, liver or kidney (Supplementary Figure S3A). Furthermore, we determined that *H-Pgds* upregulation in response to HFD was macrophage-specific by profiling ATMs isolated from mice fed a HFD for 1 or 6 months (Supplementary Figure S3B). Finally, *H-Pgds* was found to be upregulated in the scWAT as well as the epididymal WAT of ob/ob mice (Supplementary Figure S4A).

### *H-Pgds* is upregulated during monocyte to macrophage differentiation

It was striking that *H-Pgds* was regulated in a biphasic manner in ATMs isolated from 5- and 16-week-old ob/ob mice compared with WT. As mentioned above, we have previously detected this phenotype for multiple markers of macrophage polarisation^40^ and again confirmed it in this study (Figure 3a). We next sought to determine whether changes in PGD_2_ levels had functional consequences in a BMDM cell model.

**Figure 4:**
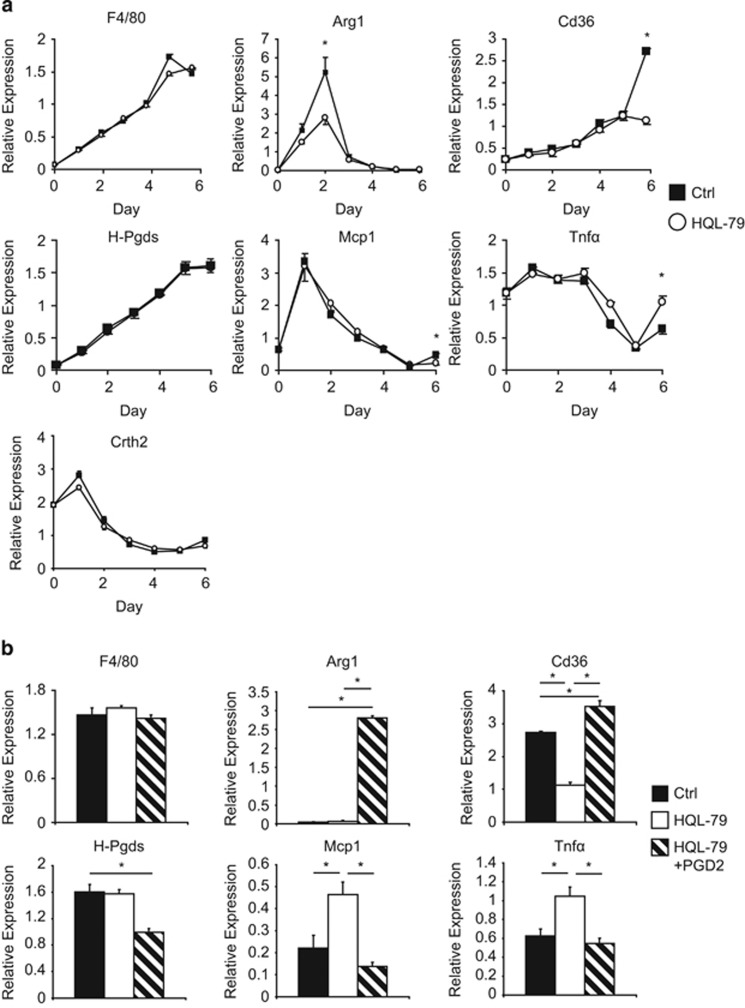
Effects of PGD_2_ on macrophage differentiation. (**a**) Expression of macrophage differentiation markers and macrophage polarisation markers during monocyte (day 0) to macrophage (day 6) differentiation. (**b**) Levels of macrophage differentiation and polarisation markers at day 6 of differentiation from BMDMs treated with either HQL-79 or HQL-79 and PGD_2_ during differentiation. *N*=4 separate BMDM cultures derived from 12-week-old C57Bl/6 male mice. *P<0.05.

We detected that *H-Pgds* was upregulated (80-fold) during differentiation from monocytes to macrophages on an mRNA level (Figure 4a). However, treatment of monocytes with the H-PGDS inhibitor HQL-79 during differentiation did not grossly inhibit macrophage differentiation, with no differences in the expression of the pan-macrophage marker *F4/80* during differentiation (Figure 4a).

### *H-Pgds* affects the terminal differentiation phenotype of macrophages

In response to treatment with HQL-79, there were subtle but significant changes in the expression of polarisation markers. Time courses for the M2 markers *Cd36* and *Arginase 1* revealed that inhibition of H-PGDS was associated with a reduction in their expression (Figure 4a) on an mRNA level. Conversely, the M1 markers *Mcp1* and *Tnfα* were significantly upregulated by inhibition of H-PGDS with HQL-79. These results suggested that endogenously produced PGD_2_ could polarise macrophages toward an M2 phenotype during differentiation (Figure 4a).

### PGD_2_ reverses the effects of inhibition of H-PGDS

To confirm that effects of HQL-79 were due to inhibition of H-PGDS, we sought to rescue inhibition of H-PGDS by administering exogenous PGD_2_. Treating BMDMs with PGD_2_ and HQL-79 simultaneously reversed the effects of H-PGDS inhibition on the expression of M1 markers (Figure 4b). Of the two PGD_2_ receptors, only *Crth2* was detectable in adipose tissue or BMDMs, suggesting that effects of PGD_2_ were mediated via CRTH2 and not the DP1 receptor (Figure 4a). Overall, these results confirmed a role for PGD_2_ in the polarisation of macrophages toward an M2 phenotype.

### Inhibition of H-PGDS during differentiation renders macrophages more prone to M1 polarisation

**Figure 5:**
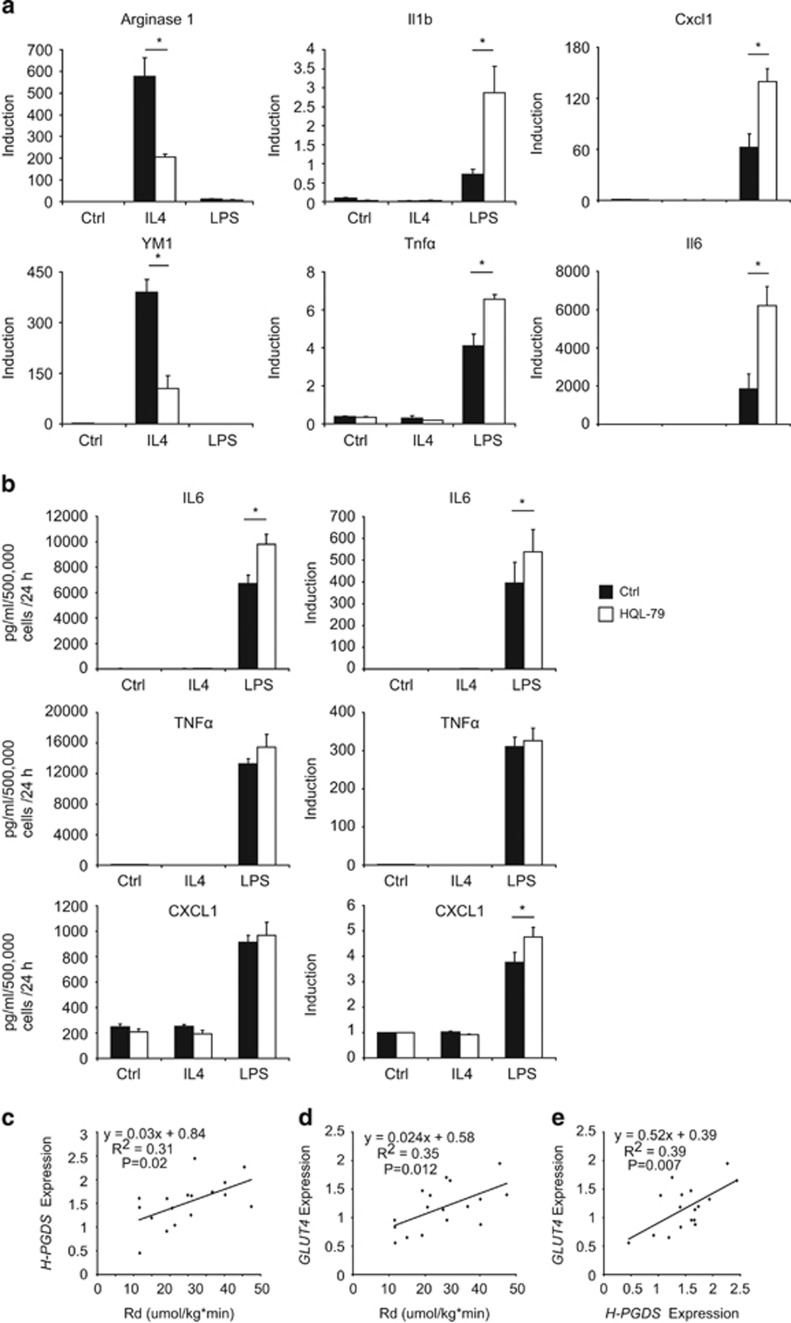
Effects of PGD2 on macrophage polarisation capacity. (**a**) Expression of polarisation markers in macrophages, treated with HQL-79 or vehicle during differentiation, in response to treatment with lipopolysaccharide (LPS) or interleukin-4 (IL4) for 24 h. (**b**) Cytokine production expressed as absolute cytokine levels (left panels) and induction from baseline (right panels). *N*=4 separate BMDM cultures derived from 12-week-old C57Bl/6 male mice. (**c**) Correlations of *H-PGDS* expression in macrophages isolated from subcutaneous WAT (scWAT) with peripheral insulin sensitivity in humans. (**d**) Correlations of *GLUT4* expression in adipocytes from scWAT with peripheral insulin sensitivity in humans. (**e**) Correlation of *H-PGDS* expression in macrophages from scWAT with *GLUT4* expression in adipocytes from scWAT in humans. *N*=17 subjects. Rd=rate of glucose disposal. *P<0.05.

Macrophages treated with HQL-79 during differentiation were subsequently treated with interleukin-4 or lipopolysaccharide. After 24 h, lipopolysaccharide induced the M1 polarisation markers *Tnfα*, *Il1b*, *Il6* and *Cxcl1* to a greater degree in HQL-79-treated cells than controls. Conversely, interleukin-4, which drives polarisation of macrophages towards an M2 phenotype, caused a significantly smaller induction in the levels of *Arginase 1* and *Ym1* in HQL-79-treated cells than in controls (Figures 5a and b).

### *Crth2* is downregulated in obese ATMs and mediates PGD2 signalling *in vitro*

At 16 weeks, ob/ob mice are profoundly obese and diabetic and express large amounts of inflammatory markers in WAT. Therefore, the finding that PGD_2_ appeared to render macrophages less inflammatory appeared to contradict with the increased levels of PGD_2_ and *H-Pgds* expression in 16-week-old ob/ob mice. However, the PGD_2_-receptor *Crth2* was downregulated sixfold in the ATMs of ob/ob mice at 16 weeks (Figure 3b), compared with a substantially smaller (twofold) induction in *H-Pgds* expression in ATMs and a twofold induction of PGD_2_ concentrations in adipose tissue. To confirm a role for *Crth2* in macrophage polarisation, BMDMs were treated during differentiation with Ramatroban, a CRTH2 inhibitor. Similar to the effects of HQL-79, Ramatroban polarised macrophages towards a more M1 phenotype (Supplementary Figure S4B).

### *H-PGDS* expression in ATMs is positively correlated with peripheral insulin sensitivity in humans

In line with the finding that PGD_2_ promotes an anti-inflammatory M2-polarisation phenotype in macrophages, the expression of *H-PGDS* in macrophages isolated from human scWAT was positively correlated with the rate of glucose disposal (Rd) in a euglycaemic clamp in 17 morbidly obese individuals (Figure 5c). Although Rd is considered a good marker of muscle insulin sensitivity, its association with adipose tissue insulin sensitivity is less clear. *GLUT4* expression in adipose tissue has been shown to be well correlated with adipose tissue insulin sensitivity.^41^ In line with this concept, we found that adipocyte *GLUT4* expression correlated with both Rd and *H-PGDS* expression in ATMs (Figures 5d and e).

## Discussion

In both dietary and genetic models of obesity, only PGD_2_ showed substantial upregulation in obese adipose tissue. Furthermore, in line with previous reports, PGE_2_ showed significant downregulation in dietary obesity^14, 15, 42^ but not in genetic obesity. Given the known inflammatory changes that occur in adipose tissue in response to dietary and genetic obesity^22, 23, 24, 25^ as well as the defined roles for many prostaglandins in inflammation,^43^ it was surprising that only PGD_2_ was associated with markers of inflammatory gene expression. It was notable that the effects on PGD_2_ concentrations and H-PGDS expression (Figures 1e and f) were much more powerful in the ob/ob mouse model than in the HFD mice. This was most likely because we used female mice, which are relatively protected from the HFD (gaining only 4 g of weight relative to WT controls); however, a role for leptin signalling cannot be excluded.

Although previous studies have investigated prostaglandin levels in whole tissue, we sought to determine which cells were producing given mediators in order to provide more information about their potential roles within WAT. The most direct approach would have been to measure lipid mediators in mechanically separated cellular fractions. This approach has two major limitations. First, a very large number of animals would be needed to obtain sufficient macrophages, adipocytes and stromal vascular cells to measure eicosanoids. The second issue, however, was more fundamental. Isolation of cells from adipose tissue by collagenase digestion is known to induce considerable stress^44^ and almost certainly would alter the endogenous prostaglandin profiles of the cells.

Although measuring eicosanoids from cellular fractions may have provided information regarding the capacity for cells to produce prostaglandins, it would not have provided information about the endogenous levels of these molecules. To avoid the issue of isolation-induced cellular stress, we applied a mathematical-based approach. By combining the concentrations of prostaglandins and the expression of mRNA markers from the same tissue samples using a PCA model, we determined that PGD_2_ was associated with macrophage markers. PGI_2_ was shown to be associated with markers of adipogenesis, a process in which it has previously shown to be a positive regulator.^2, 3, 28, 29^ Our analyses also revealed that, while correlation does not imply causation, PGE_2_ was negatively associated with lipolytic markers consistent with its known antilipolytic role.^4, 5^ Using a second multivariate statistical analysis based on hierarchical clustering of metabolites and transcriptional markers, we confirmed a strong association between PGD_2_ and the macrophage compartment. Using mRNA samples from sub-fractionated WAT, we confirmed that *H-Pgds* was predominantly expressed in macrophages and regulated in a manner consistent with a specific role in macrophage polarisation. Although it is important to note that mRNA levels may not directly equate to enzymatic activity, overall our approach provides a valuable method to avoid stress-induced changes in short-lived metabolite concentrations.

Given the expression profile of *H-Pgds*, we hypothesised that PGD_2_ would affect macrophage polarisation. As expected, blocking endogenous PGD_2_ production with the inhibitor HQL-79 affected polarisation; however, it appeared that PGD_2_ was responsible for driving macrophages toward an M2 phenotype. The increased concentrations of PGD_2_ in adipose tissue and the elevated *H-Pgds* expression in 16-week-old ob/ob macrophages seemed inconsistent with an anti-inflammatory role for PGD_2_. However, the PGD_2_ receptor *Crth2* was much more potently downregulated in macrophages than *H-Pgds* was upregulated, suggesting that PGD_2_ signalling may be compromised in obese, insulin-resistant states. Furthermore, inhibiting CRTH2 in BMDMs polarised them towards a more M1 phenotype. Finally, our results suggesting a protective role for *H-Pgds* on macrophage polarisation were further supported by the fact mice transgenically overexpressing *H-Pgds* are more insulin sensitive.^45^

Finally, to investigate a potential role for *H-PGDS* in human macrophage function, we used ATMs isolated from human scWAT. We found that *H-PGDS* mRNA levels correlated with both *GLUT4* expression in adipocytes (a marker of adipose tissue insulin sensitivity) and peripheral insulin sensitivity. Although our results are promising, further investigation into the role of *H-PGDS* in human adipose tissue will be required. Expression of M2 markers in human subcutaneous ATMs have previously been shown to correlate with insulin sensitivity in a similar manner.^46^

As part of our study, we attempted to separate the loading of substrates for eicosanoid biosynthesis, which are present at high levels in HFD, from the effects of high-fat feeding on insulin sensitivity by using a short- and long-term high-fat feeding protocols. Surprisingly, we found almost no differences on either the metabolite or mRNA expression level between the two paradigms. This suggests that, at least for adipose tissue, feeding HFD from weaning until 16 weeks of age (13 weeks of HFD) has a similar effect to feeding HFD from 12 weeks to 16 weeks of age (4 weeks of HFD).

Overall, our study provides solid evidence for *H-Pgds* having a role in the control of ATM polarisation and obesity-associated adipose tissue dysfunction and suggests that modulation of the *H-Pgds* signalling pathway in macrophages may, in future, be a promising approach for the treatment of insulin resistance and diabetes. To obtain these conclusions, it has been necessary to develop a new technique to allow the assignation of the origin of metabolites to specific cell types within tissues without the need to isolate individual cells. This method has multiple potential applications for the study of tissues where isolation and profiling of cellular fractions is either impossible or in states where the isolation procedure itself will cause unwanted alterations in metabolite levels.
