# Supplementary material for: Prostaglandin profiling reveals a role for haematopoietic prostaglandin D synthase in adipose tissue macrophage polarisation in mice and humans
Source: Int J Obes (Lond). 2015 Apr 21;39(7):1151–60. doi: 10.1038/ijo.2015.34 (PMC4486370; doi:10.1038/ijo.2015.34)
Supplement: Supplementary Information 1 [file ijo201534x1.doc]

**Supplemental Figures:**

**
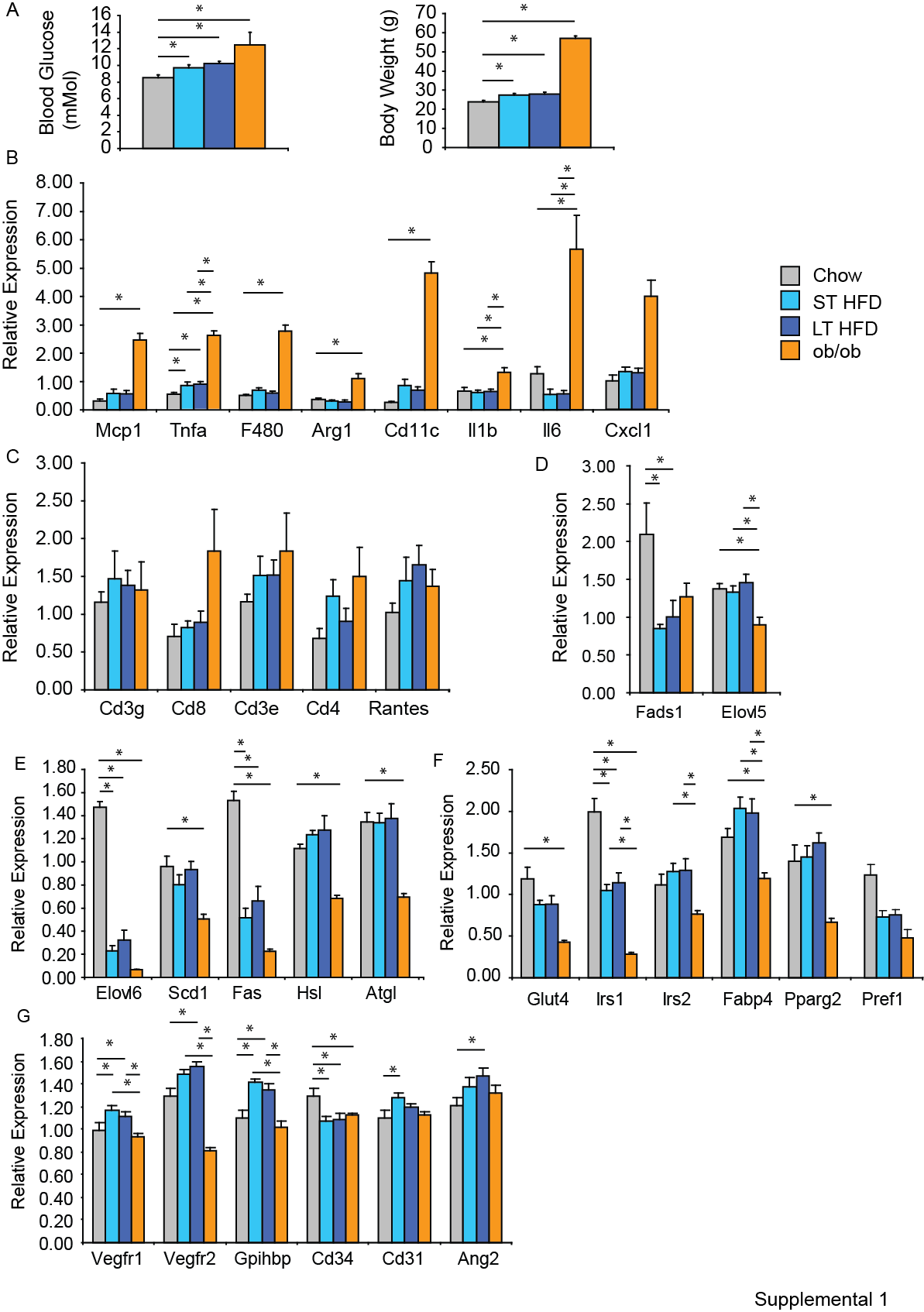
**

**
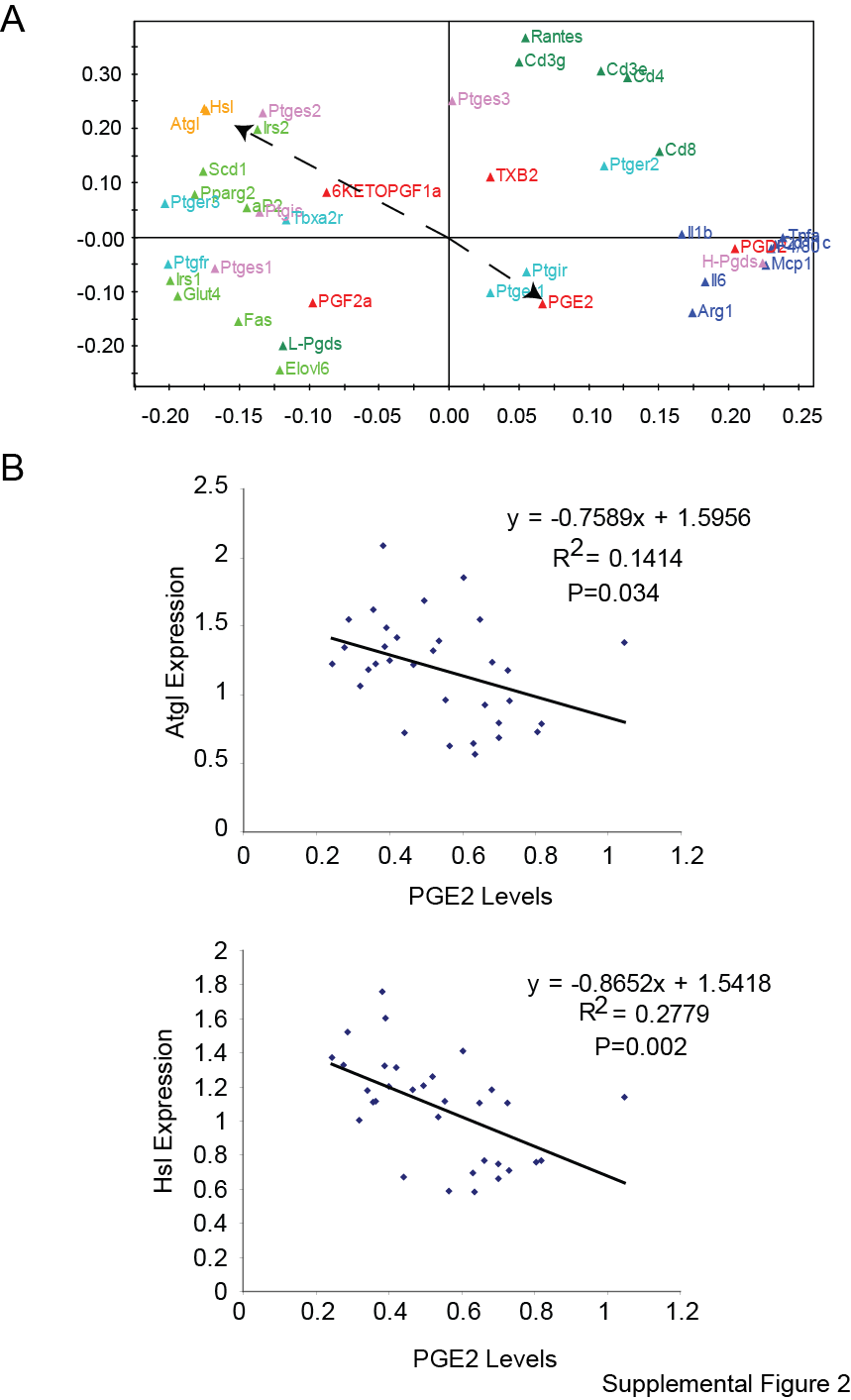
**

**
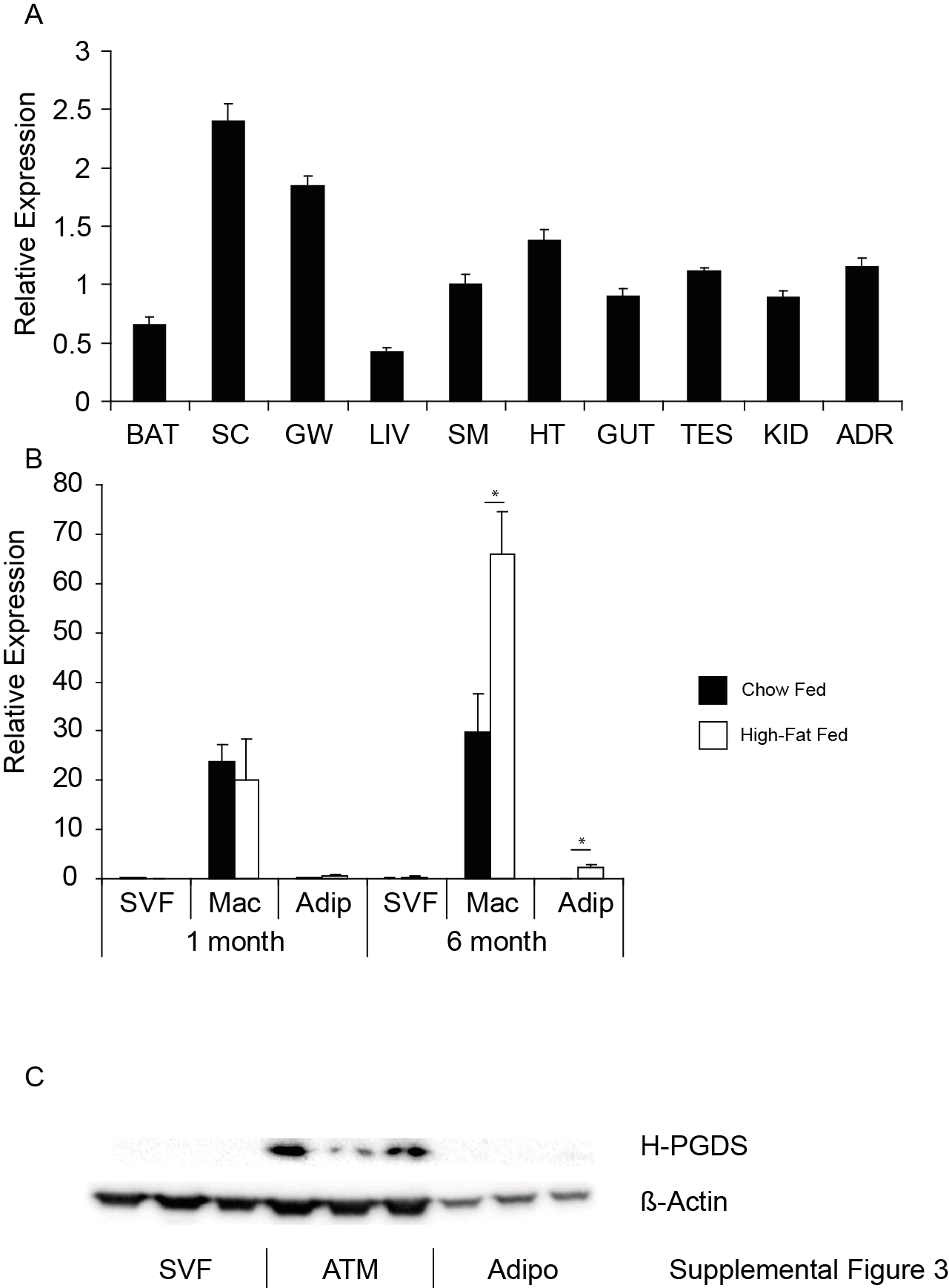
**

**
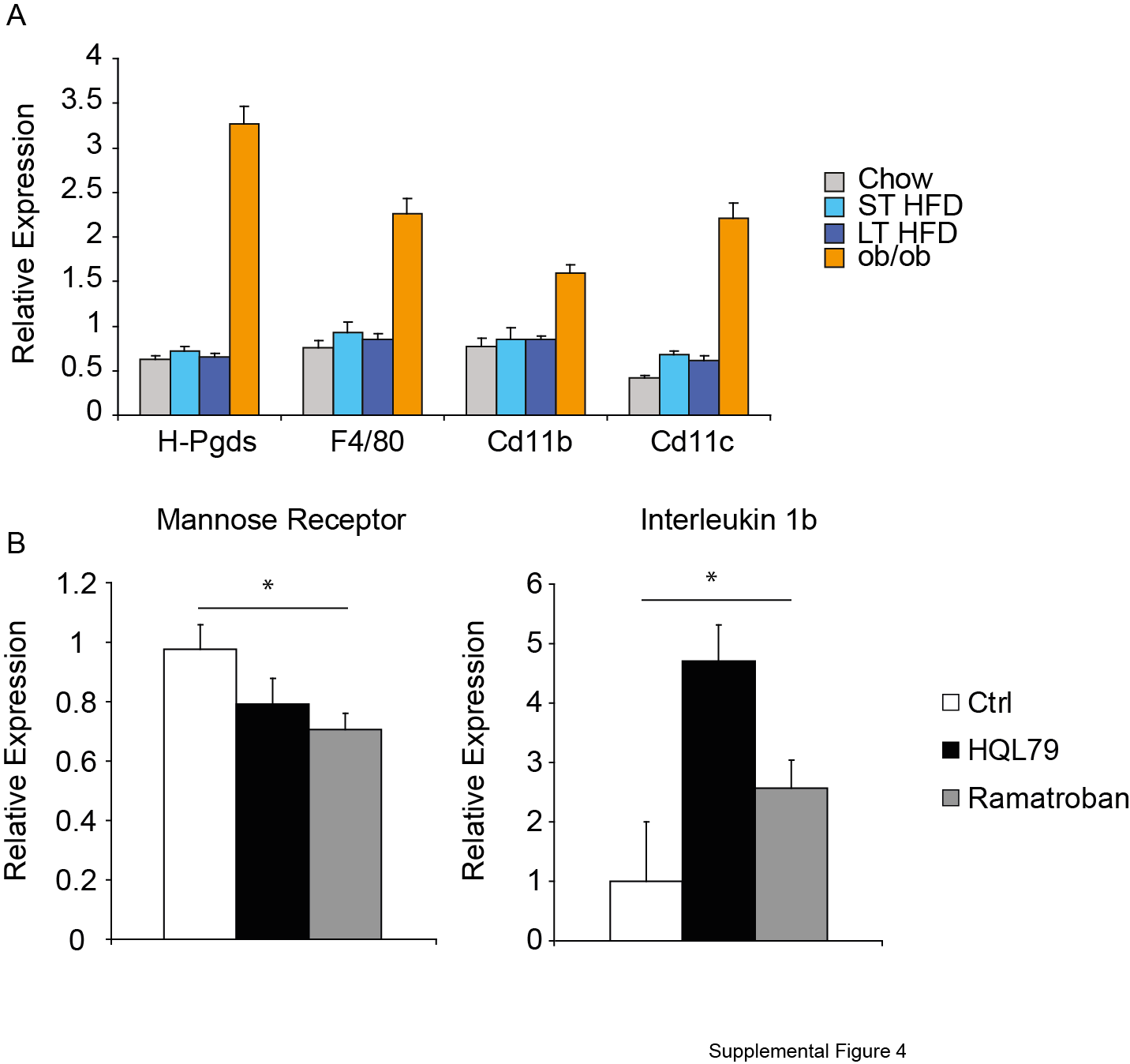
**

**
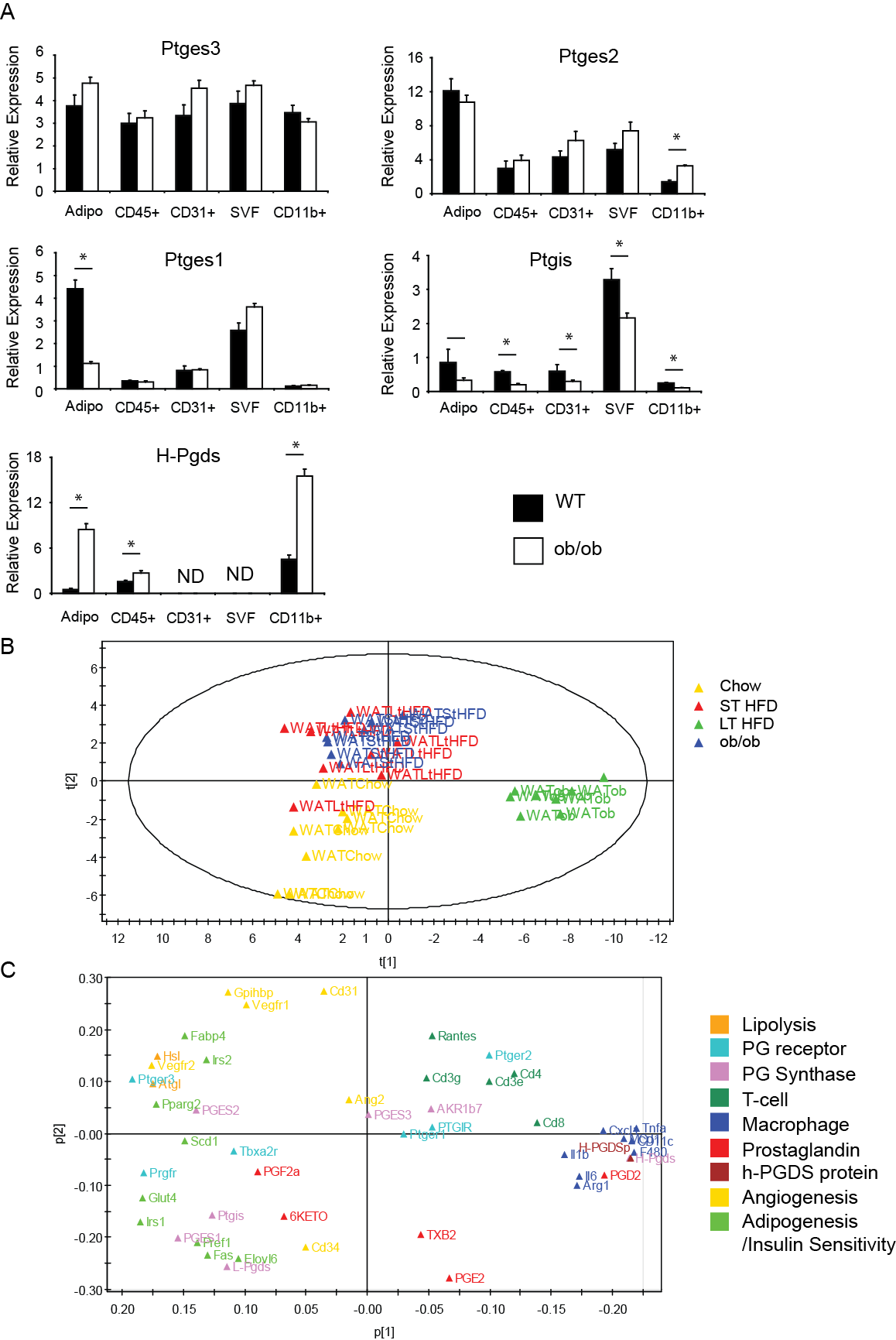
**

**Supplemental Figure5**

**
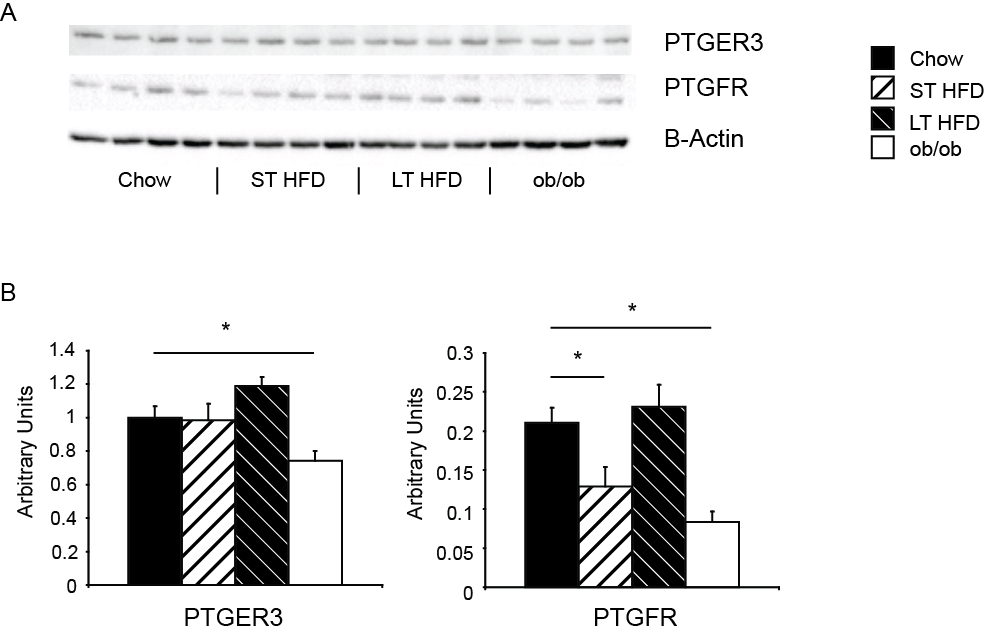
**

**Supplemental Figure 6**

**Supplemental Figure 1**A) Body weights and blood glucose levels of mice used for measurements of prostaglandins and gene expression.. Expression from perigonadal WAT of B) Macrophage markers and cytokines C) T-cell markers D) essentially fatty acid remodelling enzymes E) DNL and lipolytic genes F) markers of adipogenesis and insulin sensitivity. N=8 mice per group, C57Bl/6 females, 16 weeks of age. Chow = mice fed chow from weaning at 3 weeks. ST HFD, mice fed chow from weaning then fed HFD for 4 weeks from 12 to 16 weeks of age. LT HFD = mice fed HFD from weaning at 3 weeks of age until 16 weeks of age. ob/ob = leptin deficient ob/ob mice fed chow from weaning at 3 weeks of age until 16 weeks of age.

**Supplemental Figure 2.**

A) Loading plot for PCA analysis showing directions of lipolytic markers and PGE_2_. B) Correlation between PGE_2_ levels and *Hsl* and *Atgl* expression. N=8 mice per group, C57Bl/6 females, 16 weeks of age. Chow = mice fed chow from weaning at 3 weeks. ST HFD, mice fed chow from weaning then fed HFD for 4 weeks from 12 to 16 weeks of age. LT HFD = mice fed HFD from weaning at 3 weeks of age until 16 weeks of age. ob/ob = leptin deficient ob/ob mice fed chow from weaning at 3 weeks of age until 16 weeks of age.
.

**Supplemental Figure 3**

A) *H-Pgds* expression in different metabolically relevant tissues (n=8, female, C57/bl6 mice 10 weeks old) B) Expression of H*-Pgds*  in ATMs from perigonadal WAT, C57Bl/6 mice fed either chow or high fat diet from 8 weeks of age for the time indicated. N=3 groups of male mice for each age and diet. Black, bars, chow fed mice, white bars, high-fat fed. C) Western blot for H-PGDS from isolated stromovascular fraction (SVF) (Cd11b- fraction), Adipose tissue macrophages (ATM) (Cd11b+ fraction) and adipocytes (Adipo). N=3 groups of 4 mice, chow fed, male, C57/Bl6 mice 16 weeks of age. BAT, Brown adipose tissue; SC, subcutaneous adipose tissue; GW, gonadal white adipose tissue; LIV, Liver; SM, skeletal muscle; HT, Heart; TES, testis; KID, kidney; ADR, adrenal gland.

**Supplemental Figure 4**

A) Expression of *H-Pgds* and macrophage markers in scWAT**.** N=8 mice per group, C57Bl/6 females, 16 weeks of age. B) Expression of macrophage polarisation markers in macrophages treated with HQL-79, Ramatroban or vehicle during differentiation.
N=4 separate BMDM cultures derived from 12 week old C57Bl/6 male mice. Chow = mice fed chow from weaning at 3 weeks. ST HFD, mice fed chow from weaning then fed HFD for 4 weeks from 12 to 16 weeks of age. LT HFD = mice fed HFD from weaning at 3 weeks of age until 16 weeks of age. ob/ob = leptin deficient ob/ob mice fed chow from weaning at 3 weeks of age until 16 weeks of age.

**Supplemental Figure 5**

A) Expression of prostgalndin synthases in subfractionated perigonadal WAT including Cd31+ and Cd45+/Cd11b- fractions. N=5 WT and N=9 ob/ob, C57Bl/6 females, 16 weeks of age.

B) PCA plot showing separation of WT, HFD fed and ob/ob mice based on prostaglandins and gene expression markers including H-PGDS protein levels and angiogenic markers. C) Loadings plot for PCA analysis shown above. N=8 mice per group, for PCA analyses. N=8 mice per group, C57Bl/6 females, 16 weeks of age. Chow = mice fed chow from weaning at 3 weeks. ST HFD, mice fed chow from weaning then fed HFD for 4 weeks from 12 to 16 weeks of age. LT HFD = mice fed HFD from weaning at 3 weeks of age until 16 weeks of age. ob/ob = leptin deficient ob/ob mice fed chow from weaning at 3 weeks of age until 16 weeks of age.

**Supplemental Figure 6**

A) Representative western-blots of PTGFR and PTGER3 B) Quantification of protein in all samples normalised to β-actin. N=8 mice per group, C57Bl/6 females, 16 weeks of age. Chow, 16 week old mice fed a chow diet from weaning, ST HFD, mice fed a high-fat diet from 12 to 16 weeks of age, LT HFD, mice fed a high-fat diet from weaning until 16 weeks of age, ob/ob, leptin-deficient mice fed a chow diet from weaning until 16 weeks of age.

Supplemental table 1

| Metabolites |  |
| --- | --- |
| 6Keto | 6-Keto Prostaglandin F1α - a surogate for Prostaglandin I2 |
| PGD2 | Prostaglandin D2 |
| PGE2 | Prostaglandin E2 |
| PGF2α | Prostaglandin F2α |
| TXB2 | Thromboxane B2 - a surrogate for Thromboxane A2 |
|  |  |
| Prostaglandin Synthases | |
| *L-Pgds* | Lipocalin Prostaglandin D synthase |
| *H-Pgds* | Hematopoietic Prostaglandin D synthase |
| *Ptgis* | Prostaglandin I synthase |
| *Ptges1* | Prostaglandin E synthase 1 |
| *Ptges2* | Prostaglandin E synthase 2 |
| *Ptges3* | Prostaglandin E synthase 3 |
| *Akr1b7* | Aldo keto reductase 1b7 |
|  |  |
| Prostaglandin Receptors | |
| *Tbxa2r* | Thromboxane A2 receptor |
| *Ptger3* | Prostaglandin E receptor 3 |
| *Ptger2* | Prostaglandin E receptor 2 |
| *Ptger1* | Prostaglandin E receptor 1 |
| *Ptgir* | Prostaglandin I receptor |
| *Ptgfr* | Prostaglandin F receptor |
| *Crth2* | chemoattractant receptor-homologous molecule expressed on TH2 cells - a receptor for PGD2 |

**Supplemental table 1**

Abreviations for prostaglandins, prostaglandin receptors and prostaglandin synthases

Supplemental Table 2

| Markers used for defining adipose tissue cell types and function | | |
| --- | --- | --- |
|  |  |  |
| Marker of: | Abbreviation | Full name |
| M1 polarised Macrophages | *CD11c* | Cluster of differentiation 11c |
| M1 polarised Macrophages | *Mcp1* | Monocyte Chemoattractant protein 1 |
| M1 polarised Macrophages | *Tnfα* | Tumor Necrosis Factor α |
| Pan macrophage | *F4/80* |  |
| Monocytes/Pan macrophage | *Cd11b* | Cluster of differentiation 11b |
| M1 polarised Macrophages | *Il1b* | Interleukin 1b |
| M1 polarised Macrophages | *Il6* | Interleukin 6 |
| M1 polarised Macrophages | *Cxcl1* | C-X-C motif ligand 1 |
| M2 Polarised macrophages | *Arg1* | Arginase 1 |
| T-Cells | *Cd3g* | Cluster of differentiation 3g |
| T-Cells | *Cd8* | Cluster of differentiation 8 |
| T-Cells | *Cd3e* | Cluster of differentiation 3e |
| T-Cells | *Cd4* | Cluster of differentiation 4 |
| T-Cells | *Rantes* | Regulated on activation, normal T cell expressed and secreted |
| Essential fatty acid biosynthesis | *Fads1* | Fatty-acid desaturase 1 |
| Essential fatty acid biosynthesis | *Elovl5* | Elongation of very long chain fatty acids 5 |
| Non-essential fatty acid biosynthesis | *Elovl6* | Elongation of very long chainfatty acids 6 |
| Non-essential fatty acid biosynthesis | *Scd1* | Steroyl-Coa Desaturase 1 |
| Non-essential fatty acid biosynthesis | *Fas* | Fatty-acid synthase |
| Lipolysis | *Hsl* | Hormone-Sensitive Lipase |
| Lipolysis | *Atgl* | Adipose Triglyceride Lipase |
| Insulin sensitivity | *Glut4* | Glucose Transporter 4 |
| Insulin sensitivity | *Irs1* | Insulin receptor substrate 1 |
| Insulin sensitivity | *Irs2* | Insulin receptor substrate 2 |
| Adipogenesis | *Fabp4* | Fatty acid binding protein 4 |
| Adipogenesis | *Pparg2* | Peroxisome proliferator activator receptor gamma 2 |
| Adipogenesis | *Pref1* | Preadipocyte factor 1 |
| Endothelial cells/Angiogenesis | *Vegfr1* | Vascular endothelial growth factor receptor 1 |
| Endothelial cells/Angiogenesis | *Vegfr2* | Vascular endothelial growth factor receptor 2 |
| Endothelial cells/Angiogenesis | *Gpihbp* | Glycosylphosphatidylinositol anchored high density lipoprotein binding protein 1 |
| Endothelial cells/Angiogenesis | *Cd34* | Cluster of differentiation 34 |
| Endothelial cells/Angiogenesis | *Cd31* | Cluster of differentiation 31 |
| Endothelial cells/Angiogenesis | *Ang2* | Angiopoietin 2 |

**Supplemental table 2**

Abbreviations for markers of white adipose tissue cell types and functions encompassing major known cell types in adipose tissue with defined, discretely expressed cell markers and major functions of adipose tissue.
