# Supplementary material for: Prostaglandin profiling reveals a role for haematopoietic prostaglandin D synthase in adipose tissue macrophage polarisation in mice and humans
Source: Int J Obes (Lond). 2015 Apr 21;39(7):1151–60. doi: 10.1038/ijo.2015.34 (PMC4486370; doi:10.1038/ijo.2015.34)
Supplement: Supplementary Information 2 [file ijo201534x2.doc]

**Methods**

***Eicosanoid isolation by solid phase extraction (SPE)***

Eicosanoids were extracted from adipose tissue as described previously[^1^](#_ENREF_1). Adipose tissue extracts were acidified with 0.1 M hydrochloric acid to pH 3.0 and immediately applied to pre-conditioned SPE cartridges (C18-E, 500 mg, 6 mL) (Phenomenex, Macclesfield, UK). The cartridges were washed with 15% (v/v) methanol in water (20 ml) followed by water (20 ml) and hexane (10 ml); the lipid mediators were eluted in methyl formate (15 ml). The organic solvent was evaporated using a fine stream of nitrogen and the remaining residue was re-dissolved in ethanol (100 μl) and stored at –20ºC awaiting analysis .

***LC-MS/MS analysis of eicosanoids*:**

Chromatographic analyses were performed using Accela UHPLC (Thermo Scientific, Hemel Hempstead, UK). The bioactive lipids were separated on a C_18_ reversed-phase (RP) LC column (Phenomenex Luna, 3 μm particles, 150x2mm) using a linear mobile phase gradient (A, 0.02% glacial acetic acid in water; B, 0.02% glacial acetic acid in acetonitrile). Mass spectrometry analyses were carried out on LTQ Velos (Thermo, Hemel Hempstead, UK) linear ion trap (LIT)-orbitrap as described previously [^2^](#_ENREF_2).

***Subcellular fractionation of adipose tissue***

Gonadal adipose tissue depots were removed after sacrifice, and then chopped thoroughly and resuspended in 10 ml digestion solution (7 ml Hanks’ Solution, 3 ml 7.5 % BSA, and 20 mg Collagenase type II, Sigma). The digestion was performed at 37 °C using a shaker at 100 rpm for 20 min. After that, the adipocyte fraction (floating) was isolated and the remaining solution was centrifuged at 1500 rpm, 4 °C for 5 min. This pellet was resuspended in 1 ml of selection buffer (PBS, 2 mM EDTA, 0.5 % BSA). CD11b positive cells were subsequently selected using CD11b micro-beads (Miltenyi Biotec) according to the manufacturer’s instructions. The negative fraction from this isolation was collected and referred to as the stromal vascular fraction (SVF). It is important to note that this method isolates CD11b+/Cd11c+ M1 macrophages, however CD11c+/Cd11b-ve dendritic cells remain part of the SVF.

## Endothelial cell isolation

Gonadal fat pads were collected the tissue from C57BL/6J mice (WT, control group) and ob/ob mice. To isolated EC from gWAT we used 5 pools1 of 4 WT mice and 9 ob/ob mice individually. Fat pads were collected into Dulbecco's Modified Eagle's medium, minced thoroughly with surgical scissors and digested at 37ºC using a shaker at 100 rpm for 20 min in digestion buffer (10 ml Hanks’ buffered salt solution, 2% BSA, 2 mg/ml Type II collagenase (Sigma). After digestion, the floating mature adipocyte fraction (top layer) was collected. The solution containing the SVF fraction was filtered through a 100 μm mesh and centrifuged at 1,500 rpm at 4°C for 5 min. The pellet was resuspended in 1 ml selection buffer (PBS, 2 mM EDTA, 0.5% BSA and 2 mM glucose) and negative selection of CD31^+^ hematopoietic cells occurred in two steps. Firstly, CD11b^+^ cells were removed using CD11b^+^ microbeads followed by removal of CD45^+^ cells using CD45^+^ microbeads, according to the manufacturer’s instructions (Miltenyi Biotec). ECs were selected by incubation of the resultant CD11b^-^/CD45^-^ fraction with biotin rat anti-mouse CD31 antibody (BD Pharmingen) at the concentration of 1 μg/ml for 15 min at 4ºC, followed by incubation with streptavidin microbeads (Miltenyi Biotec), according to the manufacturer’s instructions.

***Western blotting***

All proteins were extracted into RIPA buffer. SDS-PAGE was carried out using the Novex-NuPage Midi system with 4-12% precast gradient gels with Mops buffer (Life technologies). Proteins were transferred using the iBlot system (Life Technologies). Membranes were imaged using the ChemiDoc MP digital gel documentation system (BioRad). Antibodies used were as follows H-PGDS (LS-B2422, Source Bioscience, diluted 1:1000 in 5% BSA/TBS-T, membrane blocked with 5% marvel milk in TBS-T) PTGFR (Cayman, 101802 diluted 1:1000 1:1000 in 5% BSA/TBS-T, membrane blocked with 5% BSA in TBS-T) PTGER3 (Cayman, 101760 diluted 1:200, membrane blocked with 5% BSA in TBS-T) β-Actin ( AbCam, ab8227, dilutes 1:2000, membrane blocked with 5% BSA in TBS-T).

**RNA extraction and Real-Time PCR**

Total RNA was isolated from cells and AT fractions using Buffer RLT and purified by RNeasy Mini columns (Qiagen). RNA was isolated from ground tissues using STAT-60 reagent (TEL-TEST) according to manufacturer’s instructions. Complimentary DNA was generated from 500 ng of RNA using M-MLV reverse transcriptase (Promega) according to manufacturer’s instructions. cDNA was quantified using an ABI Prism 7900 sequence detection system (Applied Biosystems) according to manufacturer’s default protocols. Data was normalised to 18s rRNA. Primer sequences available on request

***
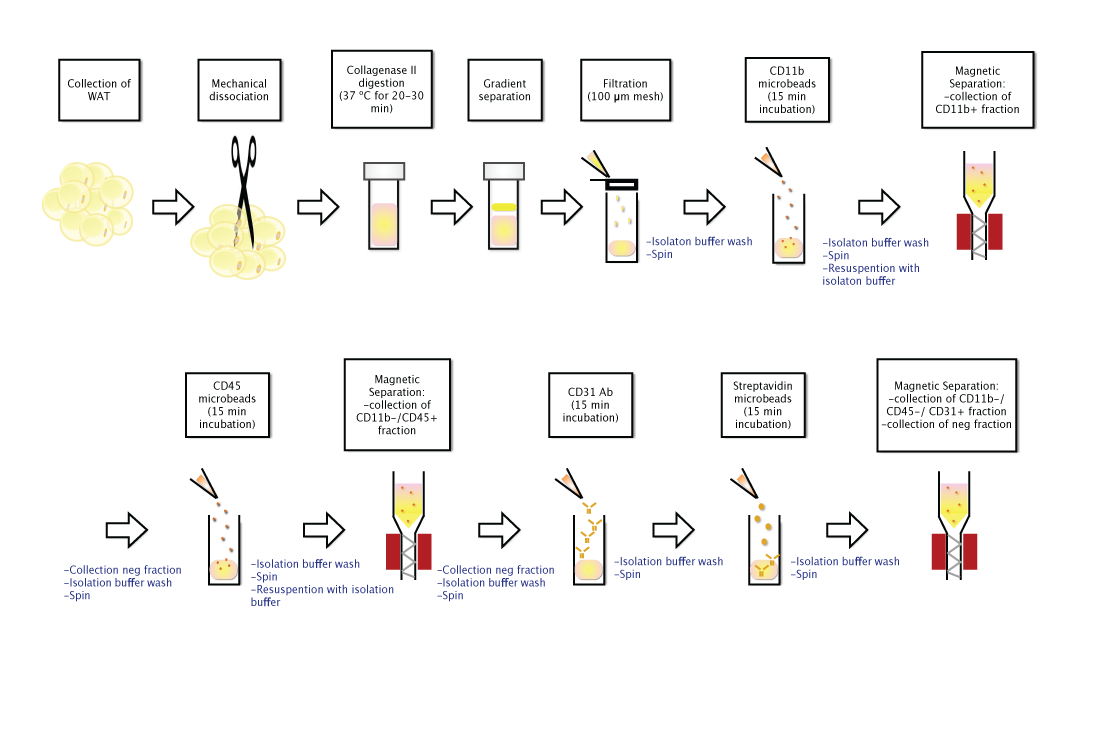
***

***Human studies***Subcutaneous adipose tissue biopsies were collected from 18 individuals undergoing bariatric bypass surgery. Macrophages were extracted as described for mice (see above) except using CD11b microbeads optimised for human cells (Miltenyi Biotec).

Prior to surgery**,** the subjects underwent a two step hyper-insulinaemic euglycaemic clamp. In one subject, the clamp was unsuccessful due to problems with the intravenous lines. Subjects were admitted to the Metabolic Clinical Research Unit of the AMC and were studied in the supine position. After a 10-h fast from 22:00 PM the day before, a catheter was inserted into the dorsal vein of the hand or distal vein of each arm. One catheter was used for sampling of arterialized blood using a heated hand box (60°C). The other catheter was used for infusion of [6,6-^2^H_2_]glucose, glucose 20%, and insulin. At T=09:00 h (T= -2), after drawing a blood sample for background enrichment of plasma glucose, a primed-continuous infusion of [6,6-^2^H_2_]glucose (99% enrichment; Cambridge Isotopes, Andover, MA) were started at a rate of 0.11 µmol/kg/min after a priming dose equivalent to 120 min infusion. After 110, 115 and 120 min, blood samples were drawn for determination of glucose enrichments, glucoregulatory hormones and FFA. Subsequently, at T=11:05 h (T=0), a continuous infusion of insulin (Actrapid 100U/ml; Novo Nordisk Farma, Alphen a/d Rijn, the Netherlands) was started for 2h at a rate of 20 mU/m^2^ body surface area min^-1^. At T=2h, the infusion rate of insulin was increased to 60mU/m^2^ body surface area. Plasma glucose was measured every 10 min and glucose 20% was infused at a variable rate to maintain plasma glucose at 5.0 mmol/L. [6,6-^2^H_2_]glucose was added to the 20% glucose solution to achieve glucose enrichments of 1% to minimize changes in isotopic enrichment due to changes in the infusion rate of exogenous glucose,. At T = 2h and T = 4h, 5h blood samples with a 5 min interval were drawn to measure glucose and enrichments and 2 samples were drawn to measure glucoregulatory hormones and FFA. During the study the participants were only allowed to drink water. Written informed consent was obtained from all subjects. The study was approved by the Institutional Review Board and conducted at the Academic Medical Center in accordance with the Declaration of Helsinki (updated version 2008). The samples are derived from a study on the short term effects of bariatric surgery on metabolic fluxes, Dutch Trial Register (NTR1548).

***Statistics – Neat Map Generation***

The average linkage algorithm was used for dendrogram generation whereas the angular positions of the profiles were determined after a dimension reduction step – again using principal component analysis – on the scaled profile data. Using this approach has two advantages: firstly the gene and metabolite profiles were oriented in way that is driven the major underlying variance components in the data (and thus displays anti-correlated behaviour more intuitively than rectangular heat maps), and secondly the similarity between gene and metabolite profiles can still be interpreted by inspecting the dendrogram structure. Metabolite and gene expression profiles were scaled to unit variance allowing a straightforward inspection of their relative changes across the phenotypic classes, which were oriented in radial direction. The clustering analysis is unable to deal with missing values and for this reason PGF2α had to be excluded due to missing values in one chow sample.

1. Masoodi M, Volmer DA. Comprehensive Quantitative Determination of PUFA-Related Bioactive Lipids for Functional Lipidomics Using High-Resolution Mass Spectrometry. *Methods Mol Biol* 2014; **1198:** 221-32.

2. Masoodi M, Eiden M, Koulman A, Spaner D, Volmer DA. Comprehensive lipidomics analysis of bioactive lipids in complex regulatory networks. *Anal Chem* 2010; **82**(19)**:** 8176-85.
